# MicroRNAs and Current Concepts on the Pathogenesis of Abdominal Aortic Aneurysm

**DOI:** 10.21470/1678-9741-2016-0050

**Published:** 2017

**Authors:** Edwaldo Edner Joviliano, Mauricio Serra Ribeiro, Emanuel Junior Ramos Tenorio

**Affiliations:** 1 Department of Surgery and Anatomy, Faculdade de Medicina de Ribeirão Preto, Universidade de São Paulo (FMRP-USP), Ribeirão Preto, SP, Brazil.

**Keywords:** Aortic Aneurysm, Aortic Aneurysm, Abdominal, MicroRNAs

## Abstract

Objective: Abdominal aortic aneurysm is an important cause of morbidity and mortality in the elderly. Currently, the only way to prevent rupture and death related to abdominal aortic aneurysms is through surgical intervention. Endovascular treatment is associated with less morbidity than conventional treatment. The formation of an aneurysm is a complex multifactorial process, involving destructive remodeling of the connective tissue around the affected segment of the aorta wall. MicroRNAs are small sequences of non-coding RNAs that control diverse cellular functions by promoting degradation or inhibition of translation of specific mRNAs. A profile aberrant expression of miRNAs has been linked to human diseases, including cardiovascular dysfunction.

**Table:** 

Abbreviations, acronyms & symbols	
AAA	= Abdominal aortic aneurysms
COPD	= Chronic obstructive pulmonary disease
EVAR	= Endovascular aneurysm repair
JNK	= c-Jun N-terminal kinase
MMPs	= Matrix metalloproteinases
SMCs	= Smooth muscle cells
TAAs	= Thoracic aortic aneurysms
TIMPs	= Tissues by metalloproteinase inhibitors

## INTRODUCTION

Abdominal aortic aneurysms (AAA) are more frequently found in clinical practice. An AAA is a permanent dilation, located in the abdominal aorta (from the level of the diaphragm and extending to its bifurcation to the right and left common iliac arteries), that exceeds the normal diameter by 50% or is larger than 3 cm. Most aneurysms are found in the infrarenal aorta, proximal to the aortic bifurcation^[1]^.

The pathological processes involved in the formation of degenerative AAA include upregulation of proteolytic pathways, apoptosis, oxidative stress, inflammation, and loss of the arterial wall matrix^[2]^. Although AAA seems to be a focal lesion, ample evidence indicates that the entire vascular system is abnormal in patients with AAA^[3]^. Molecular and biomechanical changes found in remote AAA vasculature are similar to those present in the wall of the aneurysm^[4]^.

The risk of AAA rupture increases as the diameter of the aorta increases. Mortality after rupture is high; about 80% of those who arrive at the hospital and 50% of those who undergo a ruptured AAA surgery will die. The basis for the management of AAA disease is to diagnose it before the rupture and to offer elective surgical correction at an opportune time^[5]^.

However, diagnosis is problematic since most aneurysms are asymptomatic until rupture. The significantly reduced mortality after elective repair, compared to that which occurs after rupture, has led to the development of screening programs with ultrasound. Community-based screening services have been shown to reduce mortality from AAA in men aged 65-79 years, but are not effective in women in whom the prevalence of AAA is smaller^[6]^.

### Prevalence

Most of the early studies describing the occurrence of AAA were based on findings in post-mortem examination or population-based series. Those studies estimated the prevalence of AAA could be as high as 6% in selected populations^[7]^. Aneurysms are more prevalent in white males and mortality increases with advancing age^[8]^. Subsequently, screening programs in specific populations were used to describe the epidemiology of AAA. They reported that the prevalence of AAA was between 4% and 8% in men aged 65-80 years^[9]^.

Prevalence is approximately six times higher in men than in women^[10]^. However, there is evidence to indicate that AAA prevalence among women may be gradually increasing, with women now representing one third of patients with rupture. The reason for this trend is not fully understood. One explanation is that the increase reflects a temporal change in the prevalence of smoking among women, which increased between 1950 and 1970^[11]^.

### Incidence

The average annual incidence of new AAA diagnoses in Western populations is 0.4% to 0.67%^[12]^. That incidence is ten times lower in Asian populations^[13]^. The incidence of AAA rupture is increasing. One possible explanation for this is the decrease in mortality from cardiovascular causes, thereby increasing longevity and providing insidious growth until AAA rupture. The real magnitude of the mortality associated with the rupture of the aorta is likely to be underestimated, particularly by reducing the number of post-mortem examinations^[14]^.

### Risk Factors

#### Gender

Men are at much greater risk of AAA than women. The reasons for this are unclear, but it is likely to be a function of hormonal factors, genetic susceptibility, and exposure to risk factors^[15]^. When aneurysms are discovered unruptured in women, there appears to be an association with family history^[16]^. There is no evidence to suggest that screening women for AAA is cost-effective^[17]^.

#### Family History

Family history is a risk factor independent of the development of atherosclerosis and AAA^[15]^. Several studies have reported a high prevalence of AAA among siblings of patients with AAA^[18]^. A positive family history of AAA is associated with twice the risk of those with no family history^[19]^.

The development of AAA is genetically complex. A number of familial cases and twin studies have provided strong evidence that heredity contributes to the formation of AAA^[20]^. The probability that the monozygotic twin of a person with AAA will develop an aneurysm is 24%^[21]^.

The development of AAA is unlikely to be related to a mutation of a single gene and various genetic factors are implicated. Susceptibility genes rather than occasional genetic mutations are likely to be important, particularly those that regulate the inflammatory mediators, tissue proteases and cellular biology of smooth muscle cells (SMCs) ^[22]^.

#### Heritable connective tissue disorders

Acquired connective tissue diseases are a typical reason for aortic aneurysms in more youthful patients. Roughly 20% of thoracic aortic aneurysms (TAAs) derive from disorders related with single gene mutations. In Marfan disorder, collagen cross-linking is hindered by alterations in the fibrillin-1 gene, predisposing patients to premature, severe cystic medial degeneration. More than 600 distinct mutations in the fibrillin-1 gene on chromosome 15 have been identified in patients with Marfan disorder. Fibrillin deficiency prompts dysregulation of TGF-β signaling, bringing about abnormal activation of vascular SMCs, overabundant deposits of extracellular matrix components, abundant metalloproteinase discharge, and invasion of macrophages. Marfan syndrome patients may develop thoracic aortic aneurysms, dissection, aortic valvular ineptitude, and mitral valve prolapse. Dilatation of the aortic root, which might be followed by dissection and rupture, is seen in roughly 75% of such patients. Musculoskeletal, visual, central nervous system, and pulmonary abnormalities are nonvascular sequelae of Marfan disorder^[23]^. Patients with type IV Ehlers-Danlos disorder have a defect in type III collagen production (COL3A1) that prompts hindered arterial elasticity, dissections, aneurysm formation, and arterial rupture most commonly including medium-sized arteries. Life expectancy of patients with type IV Ehlers-Danlos disorder is drastically abbreviated, with a median life expectancy of 48 years, attributable to a great extent to arterial rupture^[24]^.

When patients with generalized matrix deficiency diseases such as Marfan disorder and Ehlers-Danlos disorder are excluded, important family histories of AAA disease might be taken from 15% to 20% of patients with AAAs. The risk of AAAs in males with a first-degree relative affected by the illness is about fourfold higher than the risk in the general population, and twin-based studies estimate a heritability of roughly 60%^[25]^.

Loeys-Dietz disorder is an autosomal dominant disorder marked by mutations of TGF-β receptor 1 or 2 (TGFBR1 or TGFBR2), prompting dysregulation of TGF-β signaling. Widespread vascular dilatation and tortuosity, musculoskeletal pathology, facial dysmorphology, and skin abnormalities are characteristics of this disorder. Almost all patients present dilatation of the aortic root, and many of them go on to have dissection^[26]^.

#### Smoking Habit

Smoking is a relevant risk factor for AAA. The relative risk of AAA is 7.6 higher in smokers^[27]^. Men who smoke more than 25 cigarettes a day are at 15 times greater risk of developing AAA contrasted with men who never smoked^[28]^. Smoking presents no less than a 3.5-fold greater increase in relative risk than any other perceived AAA risk factor, and the excess prevalence related with smoking is responsible for 75% of all AAAs 4.0 cm or larger^[29]^.

The relationship between smoking and AAA increases significantly with the number of years of smoking and decreases significantly with the number of years after smoking has stopped^[30]^. In spite of the fact that discontinuing smoking is related to a decrease in the risk for AAA, people with a remote history of smoking still have a higher risk for AAA than people who have never smoked. The impact of "ever smoking" is believed to be very durable, lasting decades. The relationship of a background marked by "ever smoking" with the development of an aortic aneurysm in men is 2.5 times more prominent than the relationship of "ever smoking" with the development of coronary artery disease and is 3.5 times more prominent than the relationship of "ever smoking" with cerebrovascular disease^[31]^.

The number of cigarettes smoked every day is relevant, however, the most critical variable is the time spent smoking^[12]^. Every year of smoking increases the relative risk of AAA by 4% in all populations^[27]^. Those that keep on smoking have faster development of AAA^[32]^.

The risk of a smoker developing AAA proceeds for no less than 10 years after quitting smoking. Nevertheless, to date no causal connection has been established between smoking and training AAA. The mechanism is independent of atherosclerosis and hypotheses incorporate interruption in collagen synthesis, modified expression of metalloproteinases, and reaction to oxidative stress^[33]^.

#### Chronic Obstructive Pulmonary Disease (COPD)

An association between COPD and AAA has been reported. The prevalence of AAA among patients with COPD has been found to be 7% to 11% and most population-based screening programs report a prevalence of 4% to 6%. The association has been explained in part as a common degradation of elastic tissue. Increased elastolytic activity has been described in the serum and lungs of patients with COPD and in the serum and aorta of patients with AAA^[6]^.

#### Lipid Levels and Obesity

The relationship between the levels of plasma lipids and AAA is disputable. Elevated serum cholesterol levels (> 240 mg/dl) were related with an OR of 2.82 for AAA (95% CI 2.13 to 3.72)^[34]^. Be that as it may, a similar retrospective epidemiological review was unable to duplicate this finding; identifying a protective effect of elevated levels of HDL in serum^[35]^. This protective effect may just be a surrogate marker of cardiovascular wellbeing, as exercise is known to build HDL cholesterol^[36]^. The conceivable part of statin treatment to stabilize the progression of AAA has been investigated. Retrospective analysis of the Dutch AAA screening database suggested that statins could moderate AAA development, but this perception has not yet been demonstrated in a prospective study^[37]^.

Central obesity and AAA are independently associated. Particular anthropometric measurements, specifically waist circumference (OR 1.14, 95% CI 1.06 to 1.22) and waist-hip ratio (OR 1.22, 95% CI 1.09 to 1.37) have been independently associated with AAA in men^[38]^. A review has demonstrated that obesity (BMI > 30 kg/m^2^) was independently associated with AAA (OR 2.0, 95% CI 1.2 to 3.4)^[39]^.

#### Hypertension

Increased blood pressure is a common risk factor for AAA, yet it has a frail association^[39]^. Hypertension (systolic blood pressure > 160 mmHg, pulmonary artery diastolic pressure > 95 mmHg) is related with the risk of AAA, though just in women^[12]^. Normal hypertension has been referred to as an independent risk factor for aneurysm rupture in men and women. It mirrors the constant hemodynamic load on the aortic wall, which adds to the brittleness of the arterial wall^[40]^.

#### Diabetes

Diabetes is a risk factor for atherosclerosis, however, it appears to be protective against the development of AAA^[29]^. A meta-analysis suggested a reduced rate of AAA among diabetes patients contrasted with patients without diabetes (OR 0.65, 95% CI 0.6-0.7, *P*<0.00^[41]^. Diabetes is additionally connected with a slower growth rate in AAA^[29]^.

The proposed components for the protective effect of diabetes comprise hyperinsulinemia, hyperglycemia, and results of therapeutic agents used to treat diabetes. These agents may balance out mural thrombus, augment the stiffness of the aortic wall, and decrease systemic inflammation^[42]^ (Table 1)^[30]^.

**Table 1:** Independent risk factors for AAA ≥ 4 cm.

Risk Factor	Odds Ratio	95% CI
**Increased Risk**		
Smoking history	5.1	4.1-6.2
Family history of AAA	1.9	1.6-2.3
Older age (per 7-year interval)	1.7	1.6-1.8
Coronary artery disease	1.5	1.4-1.7
High cholesterol	1.4	1.3-1.6
COPD	1.2	1.1-1.4
Height (per 7-cm interval)	1.2	1.1-1.3
**Decreased Risk**		
Abdominal imaging within 5 years	0.8	0.7-0.9
Deep venous thrombosis	0.7	0.5-0.8
Diabetes mellitus	0.5	0.5-0.6
Black race	0.5	0.4-0.7
Female gender	0.2	0.1-0.5

AAA=abdominal aortic aneurysm; COPD=chronic obstructive pulmonary disease

#### Pathophysiology of Abdominal Aortic Aneurysm

The aorta is made out of three layers: the intima, tunica media, and adventitia. The inward layer is the innermost wall of the aorta, comprised of endothelial cells in direct contact with the blood. These cells can add to the formation of the aneurysm by generating reactive oxygen species.

The middle layer contains extracellular connective components (elastin, collagen types I and III, and proteoglycans) and SMCs composed of lamellar functional units. These units, which form the primary load-bearing structure of the healthy aorta, are depleted significantly over years or decades in the pathogenesis of AAA. In advanced stages of the disease, the rigidity of the aorta and the collagen load are dramatically increased due to the degradations of elastin. The failure happens when the residual collagen fibers, newly synthesized from the media and adventitia, cannot maintain structural integrity. The exact succession of events that prompt rupture has yet to be identified, though it unquestionably includes inflammation and proteolysis of the middle layer, leading to significant reductions in wall tensile strength^[43]^.

The adventitia is made out of interstitial collagen, fibroblasts, nerve fibers and vasa vasorum, being effectively required in the pathogenesis of AAA. The density of the vasa vasorum diminishes along the length of the aorta from the aortic root to the bifurcation^[44]^. For a considerable length of time, there has been speculation about a potential connection between the reduced density of vasa vasorum in the adventitia and the upward trend of formation of aneurysm in the distal aorta. Nevertheless, evidence of causal connection between aneurysm development and regional differences in the vascularization of the adventitia of the aorta remains uncertain. As of late, expanded inflammationdriven neovascularization has been perceived in surgical specimens acquired at the time of aneurysm repair, found more prominently in areas of aortic rupture. In spite of the fact that this neovascularization is clearly present, it is as yet dubious whether it effectively advances the movement of aneurysmal sickness with ensuing rupture, or is basically evidence of progressive wall inflammation^[45]^.

**Fig.1:**
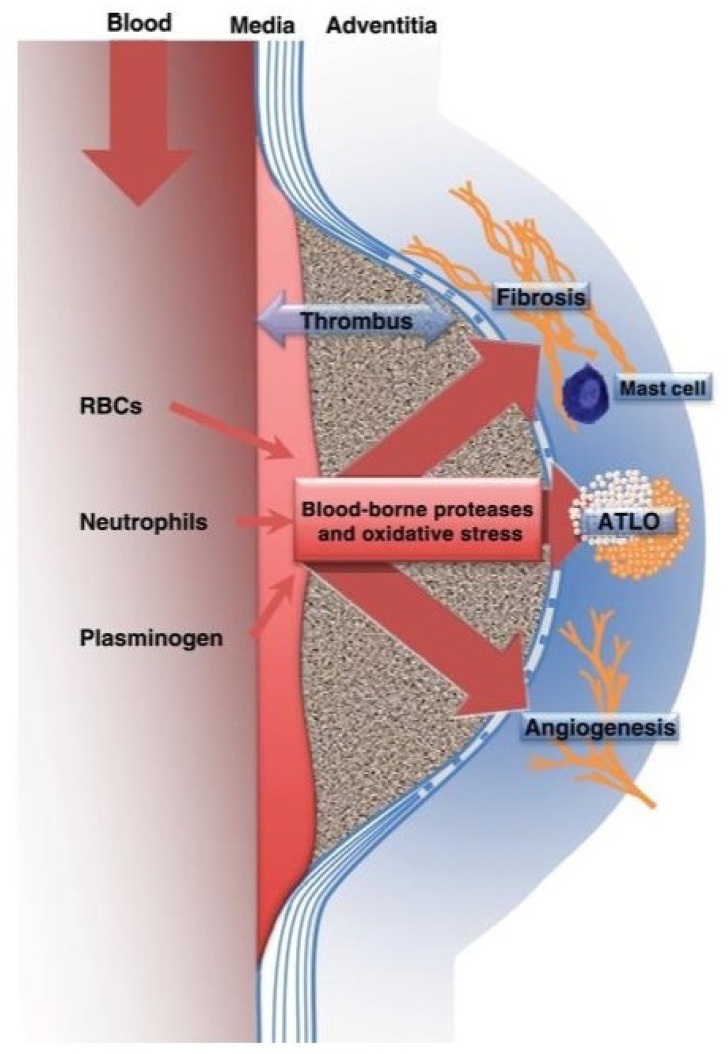
Schematic representation of the impact of the blood-chronic intraluminal thrombus interface on medial degradation and the adventitial inflammatory, angiogenic and fibrotic responses in human AAA. Abbreviations: ATLO, adventitial tertiary lymphoid organ; and RBCs, red blood cells.

The intraluminal thrombus, in addition to being a surrogate marker of illness progression, can directly intercede in the progression of AAA through proteolytic activation of matrix metalloproteinases by plasmin. Furthermore, the aggregation of thrombus may upset the dissemination of oxygen through the wall of the aorta and result in relative hypoxia potential and apoptosis/necrosis of SMCs. The laminar thrombus can likewise alter the wall voltage peak in the AAA. The impact of having a thrombus in the AAA remains unclear, yet most examiners concur that the laminar thrombus by and large expands the progression of the disease and the risk of AAA rupture of comparable diameter^[46]^ (Figure 1).

Matrix metalloproteinases (MMPs) constitute an arrangement of extracellular enzymes that degrade the cellular matrix, which is essential to an assortment of physiological processes, including homeostasis, wound healing, and tissue remodeling and resorption. All MMPs are parts of amino acid sequences that are considerably similar, containing an essential zinc ion to its enzyme activity and being inhibited by chelating agents. In addition, their inhibition in vivo happens through expression and local release of biological inhibitors of metalloproteinase activity or tissues by metalloproteinase inhibitors (TIMPs). Pro-MMPs are emitted by neutrophils, macrophages, fibroblasts, and SMCs. Activation and cleavage of the enzyme are catalyzed by extracellular proteases, such as, for example, plasmin, plasminogen activators, and other MMPs^[47]^.

MMP-9, otherwise called gelatinase B or 92 kDa gelatinase, severs elastin, collagen types I and IV, and fibrinogen. AAA disease presents higher MMP-9 plasma levels, and more MMP9 mRNA is available in the aneurysmal aortic tissue than in the typical aortic tissue. Patients with AAA of intermediate size (5 to 6.9 cm) present higher serum levels than patients with AAAs smaller than 4 cm or more prominent than 7 cm. Even though these outcomes not normalized for the volume of tissue or wall cellularity, a significant role for MMP-9 activity in disease progression can be inferred^[48]^. MMP-9 levels reliably diminish after AAA repair, both endovascular and open, and patients who continue to have high levels of MMP-9 after endovascular repair of aneurysm might be at a greater risk of developing and maintaining leaks^[49]^.

MMP-2, like MMP-9, cleaves elastin and type IV collagen. Samples taken from aortic aneurysms demonstrate that MMP-2 activity is relatively lower than that of MMP-9, which suggests that expansion in MMP-2 activity could mean an early event in the temporal evolution of AAA pathogenesis. Trial data indicate that both MMP-2 and MMP-9 should be available and active for maximum progression of the aneurysm, suggesting a synergistic and co-dependent relationship between no less than two of the most essential proteases active in AAA disease^[50]^.

Moreover, depletion of SMCs is a distinct pathological characteristic of advanced aneurysmal disease. During surgical repair, aneurysmal specimens, contrary to specimens from occlusive aortic diseases, show evidence of increased apoptosis of SMCs, including higher production of p53. The other SMCs, albeit viable, show reduced capacity for proliferation. SMCs depletion is additionally a sentinel feature of experimental aneurysm pathogenesis. A large number of experimental models have demonstrated the capacity of enhanced medial cellularity to balance out the integrity of the aorta and limit the growth of the aneurysm^[51]^.

Besides proteolysis and loss of medial cellularity, inflammation is a main pathophysiological feature of aortic aneurysm ailment. While the starting events in human AAA sickness remain unclear, there is experimental evidence indicating that elastin degradation products, or hydrophilic peptides discharged from events, stimulate and enlarge the location and activation of mononuclear cells inside the aortic wall. Elastin degradation products bind to surface proteins and provide stimulus to increase chemotaxis, phagocytosis, and activation of mononuclear cells. Patients with AAA have high serum concentrations of elastin degradation products, which is associated with the risk for sickness progression^[52]^.

Production of superoxide (O_2_-) and hydrogen peroxide (H_2_O_2_) by fibroblasts, constitutive aortic cells, and infiltrating leukocytes are examples of other early events of pro-inflammatory signaling. Higher production of these reactive oxygen species inhibits plasminogen activator inhibitor-1, an enzyme that limits MMP activation, and prompts increased proteolysis and degradation of the matrix. Compared to normal aortic tissue, AAA surgical specimens show considerably higher levels of oxidative stress^[53]^.

Inflammatory cells found in AAA surgical specimens incorporate macrophages and Th1, Th2 and B lymphocytes. There is substantial discussion about the likely differential role of Th1 and Th2 cells in mediation of aortic diseases. Despite having an overlap, recent evidence shows that Th2 cytokines responses prevail in AAA disease and that the Th1 cytokine response is normal for atherosclerotic vascular occlusive infections. Th2 lymphocytes produce interleukin-4 (IL - 4), IL - 5, IL-8 and IL - 10, which together promote wall angiogenesis, extra release of proinflammatory cytokines and apoptosis of SMCs by means of the activation of the Fas ligand. CD4+ T cells, helper T cells with receptor affinity for class II major histocompatibility complex that produce interferon - γ, are the lymphocytes most commonly found in the aortic aneurysm tissue. Mice with CD4+ T cells deficiency are impervious to the development of AAA, however, the reaction could be partially reestablished by exogenous treatment with interferon γ, while AAA formation in interferon γ deficient mice can be reconstituted by reinfusion of wild-type splenocytes^[54]^.

Numerous inflammatory responses in SMCs and macrophages incorporate kinase activation via N-terminal (JNK), otherwise called protein kinase activated by stress. JNK, a proximal signaling molecule, regulates proteolytic functions and manufactured vascular SMCs, in addition to pro-inflammatory cytokine production and proteolytic action of macrophages. To some extent, JNK activity is responsible for regulating production and activation of more than 20 proteins significant to the pathogenesis of AAA, including MMP-9 and IL-1a. JNK expression is expanded in test studies and AAA human tissue samples^[55]^.

Proteins traditionally connected with thrombosis and coagulation cascade regulation can likewise take part in the pathogenesis of AAA. One study stated that patients with aortic aneurysms had, on average, three times the protein C and activated protein C inhibitor than controls. Activated Protein C/ Protein inhibitor levels correlate with AAA diameter. Additionally, osteopontin, a pluripotent mediator of bone metabolism, inflammation and vascular calcification, may be significant in AAA. Serum and tissue osteopontin concentrations are higher in human disease, and osteopontin deficient mice demonstrate lessened AAA formation in experimental models^[56]^.

### MicroRNAs and the Formation of Aneurysms

MicroRNAs (miRNA) are single stranded RNA molecules of about 17-23 nucleotides in length, that act on the regulation of gene expression, and are involved in many cellular processes, including proliferation, differentiation and apoptosis^[57]^. Evolutionarily, its origin is of the vegetable kingdom as a way of protecting the genetic material from outside interference. The first small RNA noncoding identified was the lin-4, control gene regulator lin-14 larval growth, Caenorhabditis elegans roundworm^[58]^.

The process of miRNA biogenesis is complex and involves nuclear and cytoplasmic components^[59]^. Initially, miRNAs are produced in the form of a long primary transcript (pri-miRNA), several kilobases long, by RNA polymerase II. The pri-miRNA is processed further in the core by an RNase III endonuclease, known as Drosha, together with its cofactor Pasha, generating a mature miRNA precursor molecule termed pre-miRNA, with about 70 nucleotides. Then, the pre-miRNA is transported to the cytoplasm by exportin-5, using GTP as a cofactor.

**Fig. 2:**
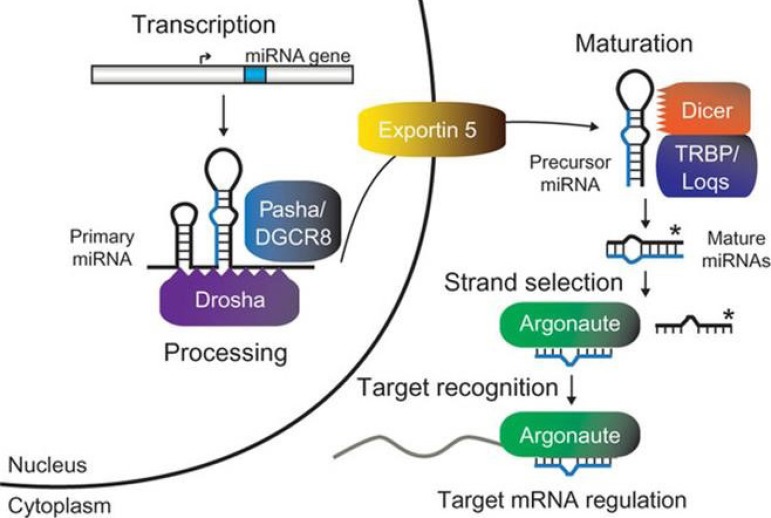
A general model of miRNA biogenesis and function.

In the cytoplasm, the pre-miRNA is processed by another RNase III, Dicer, generating a double stranded miRNA of about 22 nucleotides. This product is incorporated into the RISC multiprotein complex (RNA-induced silencing complex), whose main components are Argonaut proteins. The helicase activity causes only one of the miRNA duplex tapes to remain in the RISC complex to control post-transcriptional expression of target genes^[60]^ (Figure 2)^[61]^.

MiRNAs traditionally are a class of negative regulators of gene transcription. Each miRNA may be used for bioinformatics predictions, regular to 300 different genes, according to the complementary miRNA and mRNA gene sequence^[62]^. MiRNAs are known for two mechanisms of action: mRNA degradation and inhibition of their translation. The first mechanism occurs when there is perfect pairing between the miRNA and the target gene or when the imperfect matching results in degradation of mRNA^[63]^. The imperfect matching can interfere with the inhibition of translation through boot lock, removal of ribosomes and elongation inhibition^[64]^.

Experiments with specific SMCs emphasize the importance of miRNAs for homeostasis of vascular SMCs ^[65]^, and it is likely that miRNAs also play a role in aneurysm formation, which is characterized by dysfunction of vascular SMCs. Indeed, it has recently been shown that miRNA-29 plays a key role in the formation of aneurysms^[66]^.

These studies have reported that inhibition of miRNA-29 reduces the formation of aneurysms in various murine models. Specifically, inhibition of all miRNA-29 family has been shown to prevent angiotensin II and induce dilatation of the aorta in wild type mice^[66]^. miRNA-29b inhibitor reduced the progression of aneurysm in an elastase infusion model in porcine pancreatic C57BL6 mice and, to a lesser extent, in the angiotensin II infusion model in ApoE ^- / -^ mice^[67]^.

Similar results have been demonstrated in models using mice with genetic Marfan^(Fbn1C1039G /+)^, in which miRNA-29b locking prevented the development of aneurysm and apoptosis of the aortic wall^[68]^. Furthermore, increased expression of miRNA-29 induced severe aneurysm expansion in two different murine models^[67]^.

All these studies indicate the same molecular mechanism: miRNA-29 regulates multiple target levels of expression with a role in extracellular matrix, and therapeutic inhibition of miR-29 improves the structure and integrity of the vessel wall. It has been previously demonstrated that, in the heart, miRNA-29 acts on different targets of the extracellular matrix, such as collagen and elastin. These extracellular matrix components are also induced after inhibition of miRNA-29 in the vasculature^[66]^. Interestingly, inhibition of miRNA-29 can also be used to increase elastin expression in patients with insufficient haploidentical cells as well as elastin deposition in bioengineering vessels^[60]^. Besides acting on the structural components of the extracellular matrix, miRNA-29 also targets anti-apoptotic MCL-1 protein and, paradoxically, MMP-2. In fact, a decrease in MCL-1 protein was found in mice with Marfan, and inhibition of miRNA-29 prevented apoptosis, which may contribute to the therapeutic effects of inhibition of miRNA-29^[68]^.

**Fig. 3:**
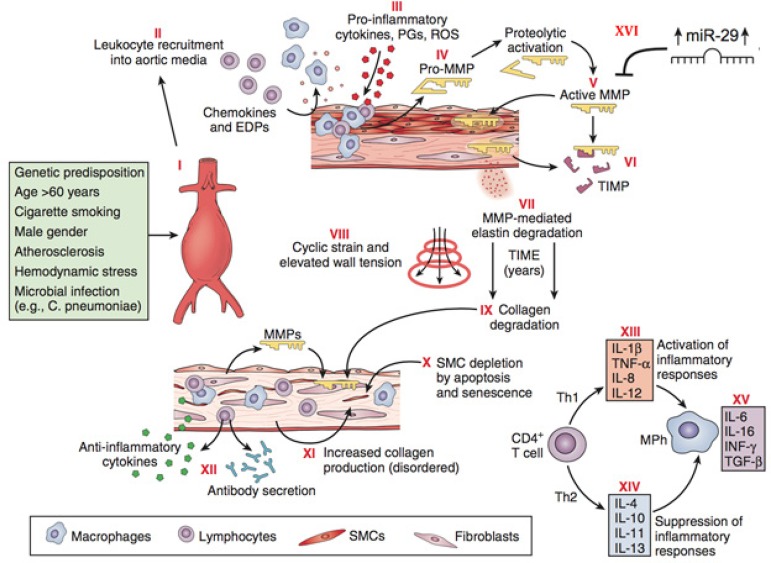
Pathophysiology of abdominal aortic aneurysms. Schematic diagram illustrating events thought to contribute to the development and progression of abdominal aortic aneurysms. Injury to the aortic wall, either as a consequence of or in association with known risk factors (I), leads to recruitment of leukocytes into the aortic media (II), macrophage (MPh) activation, and production of proinflammatory molecules (III). Macrophages also produce proenzyme forms of matrix metalloproteinases (MMPs) (pro-MMPs) (IV), which are activated in the extracellular space (V). Tissue inhibitors of matrix metalloproteinases (TIMPs) may neutralize MMP activity (VI), but this neutralization appears insufficient to prevent degradation of structural matrix proteins (elastin and interstitial collagens) (VII). Over a period of years, elastin degradation, cyclic strain, and elevated wall tension bring about progressive aortic dilatation (VIII). Collagen degradation further weakens the aortic wall (IX); although medial smooth muscle cells (SMCs) and fibroblasts might promote structural repair, apoptosis, and cellular senescence cause SMC depletion (X), and interstitial collagen appears disorganized (XI). Aneurysm tissues exhibit infiltration by T cells, B lymphocytes, plasma cells, and dendritic cells and local deposition of immunoglobulins, reflecting a cellular and humoral immune response (XII). Understanding the adaptive cellular immune response in abdominal aortic aneurysms may reveal how different T-cell subsets (i.e., helper T cell type 1 [Th1] versus Th2) interact with macro- phages to promote or suppress aneurysmal degeneration, on the basis of the local balance of proinflammatory (XIII) and anti-inflammatory (XIV) molecules. Some cytokines produced within aneurysm tissue, such as interleukin-6 (IL-6) and interferon-γ (IFN-γ), may have dual and opposing functions depending on the specific context (XV). The promotion of miR-29 induces the extracellular matrix degradation and induces the formation of aneurysms (XVI). EDPs, Elastin degradation peptides; PGs, prostaglandins; ROS, reactive oxygen species; TGF-β, transforming growth factor-β; TNF-α, tumor necrosis factor-α; miR-29, microRNA-29^[70]^.

In human aneurysms, miRNA-29b (but not miRNA-29a and miRNA-29c) showed high expression in thoracic aneurysms in one study whereas, in another study, it was not regulated and showed low expression in abdominal aortic aneurysms (Figure 3). A recent additional report describes the association of altered levels miRNA-29 with aneurysm formation in human thoracic aneurysm zones and, using a bioinformatics approach, miRNA-29 has been proposed to contribute to aneurysm formation^[69]^ (Figure 3)^[66,70]^.

Similar to miRNA-29, other miRNAs have been described to play a role in the pathogenesis of aneurysm. One of the first reports referring to the role of miRNAs in the formation of aneurysms describes the mechanism by which miRNA-143/145 regulates SMCs function. The authors showed that, in human thoracic aneurysms, miRNA-143 and miRNA-145 were expressed at lower levels in comparison with healthy thoracic aorta, which correlated with the SMCs function. Another study points to the role of miRNA-143/145 in the maintenance of SMCs function and describes the specific elimination of SMCs in rats, leading to contractile dysfunction of SMCs that can be rescued by restoration of miRNA-143/145^[65]^. Whenever there was restoration of miRNA-143/145, aneurysm formation was not reported. Another major regulator of vascular smooth muscle phenotype cells, miRNA-21 was shown to inhibit the formation of aneurysms in rats. Overexpression of miRNA-21 protected against progression of the aneurysm while inhibition of miRNA-21 further increased the current aneurysm formation^[66]^.

### Risk of Rupture

Several factors are associated with AAA rupture. The most commonly used indicator of AAA rupture is the maximum diameter of the aneurysm (Table 2)^[71]^. Significant AAA diameter at break was 5 cm in women and 6 cm in men. After adjusting for age, initial AAA diameter, height, and BMI, the AAA rupture rate was three times higher in women than in men. Therefore, diameter limits AAA^[39]^.

**Table 2:** Estimated annual rupture risk.

AAA diameter (cm)	Rupture risk (%/y)
< 4	0
4 – 5	0.5 – 5
5 – 6	3 – 15
6 – 7	10 – 20
7 – 8	20 – 40
> 8	30 – 50

AAA=abdominal aortic aneurysm

Studies with biomarkers and biomechanics were investigated for their usefulness in predicting AAA rupture and, although specific finite element programs are promising, these techniques are not sufficiently specific to be applied in clinical practice^[72]^. Studies examining putative biomarkers for AAA rupture are scarce and have focused on plasma proteins sensitive to inflammation, such as fibrinogen and α-1-antitrypsin^[73]^. Increased levels of these markers are likely to be a consequence of the rupture rather than a real risk predictor^[9]^.

AAA growth was suggested to have an independent effect on the risk of rupture. A growth greater than 1 cm per year has been used as an independent indication for AAA surgery^[9]^. Other factors have been shown to be independent and significantly associated with the risk of AAA rupture, namely: female gender (OR 4.5, 95% CI 1.98 to 10.2), smoking (OR 2.1, 95% CI 0.95 to 4.67), and hypertension (mean blood pressure > 110 mmHg) (OR 1.04, 95% CI 1.02 to 1.07)^[39]^. Pharmacological mechanisms to reduce the risk of AAA rupture have been explored. One study showed that patients taking angiotensin converting enzyme inhibitors are less likely to present with AAA rupture (OR 0.82, 95% CI 0.74- 9)^[74]^.

### Treatment and Follow-up

Once an aneurysm is established, its natural history grows gradually. A clearer understanding of the AAA pathophysiology led to testing pharmacological strategies to limit the expansion of the aneurysm. β-blockers and antibiotics have failed to translate the results in animal studies to therapies in clinical practice^[75]^. At present, surgery is the only proven effective treatment to prevent rupture of AAA and death related to the aneurysm. This intervention should be carried out when the AAA reaches 5.5 cm in diameter in male patients and 5 cm in female patients and in all symptomatic aneurysms, regardless of diameter.

Initial evidence of the benefit of endovascular aneurysm repair (EVAR) over open surgery was provided by single-center and registry data. These registries described 2.9% to 5.8% mortality for elective EVAR and led to randomized controlled trials comparing established open surgery with endovascular techniques. Three randomized controlled trials have compared EVAR with open surgery in patients fit for elective surgery. The EVAR1, DREAM, and OVER trials have reported medium-term and long-term outcomes. All studies have demonstrated an early perioperative mortality benefit for EVAR *versus* open surgery (EVAR1: 1.7% *versus* 4.7%, *P*=0.009; DREAM: 1.2% *versus* 4.6%, *P*=0.1; OVER: 0.5% *versus* 3.0%, *P*=0.004). In addition, patients assigned to EVAR had less blood loss, required fewer blood transfusions, and had reduced intensive-care stay than patients assigned to open surgery. However, no difference between the two treatment options was found for long-term (>2 years) total mortality or AAA-related mortality^[9]^.

The uptake of EVAR for elective surgical management of AAA is now approaching 80% in many centers. In the context of surgical management of a ruptured AAA, a substantial body of evidence demonstrates improved survival with an EVAR-first approach^[9]^.

Leak rates range from 0 to 47%, depending on the type of stent graft, patient selection, implantation technique and morphology of the aorta. The presence of leaks can be associated with further expansion of the aneurysm, which in turn may result in rupture. Thus, it becomes necessary to monitor patients submitted to endovascular repair of AAAs using computed tomography scans, with a significant increase in costs of the overall process^[76]^.

## CONCLUSION

In conclusion, it can be said that the formation of an aneurysm is a multifactorial complex process, involving the destructive remodeling of the connective tissue around the affected segment of the aortic wall. In recent years, considerable effort has been dedicated to elucidate the molecular mechanisms and AAA training roads, with recent studies focusing on the role of miRNAs. By understanding the pathophysiology of aneurysm formation, treatments with specific drugs can be designed to interrupt the growth of or even to avoid their breakage. The study of miRNAs and their modulation will add to our understanding of the formation AAA and may result in potential therapeutic targets.

**Table:** 

Authors' roles & responsibilities	
EEJ	Conception and design of the work; final approval of the version to be published
MSR	Revising it critically for important intellectual content; final approval of the version to be published
EJRT	Acquisition, analysis, and interpretation of data for the work; final approval of the version to be published
